# Corrosion Resistance of Laser Powder Bed Fused AISI 316L Stainless Steel and Effect of Direct Annealing

**DOI:** 10.3390/ma15186336

**Published:** 2022-09-13

**Authors:** Kichang Bae, Dongmin Shin, Jonghun Lee, Seohan Kim, Wookjin Lee, Ilguk Jo, Junghoon Lee

**Affiliations:** 1 Department of Metallurgical Engineering, Pukyong National University, Busan 48513, Korea; 2 Department of Materials Science and Engineering, Ångström Laboratory, Uppsala University, 75321 Uppsala, Sweden; 3 School of Materials Science and Engineering, Pusan National University, Busan 46241, Korea; 4 Advanced Materials Engineering, Dong-Eui University, Busan 47340, Korea

**Keywords:** laser powder bed fusion, selective laser melting, AISI 316L stainless steel, corrosion resistance, anisotropy

## Abstract

Alloy parts produced by an additive manufacturing method with rapid heat transfer from fast melting and solidification have different microstructures, characteristics, and performances compared with materials made by the conventional process. In this study, the corrosion and oxidation resistance of SS316L, which was prepared by the powder bed fusion process, was compared with those of cold-rolled SS316L. Additionally, the surface oxide film on stainless steel was thoroughly assessed since the film has the greatest influence on the corrosion and oxidation resistance. The effect of heat treatment on corrosion and oxidation resistance of SS316L fabricated by additive manufacturing was investigated. The SS316L has a microstructure formed by sub-grain cells, in which locally concentrated alloying elements form a stable passive film. As a result, it has a higher level of corrosion resistance and oxidation resistance than conventional cold-rolled materials. However, it was confirmed that the sub-grain cell was removed by heat treatment, which resulted in the degradation of corrosion and oxidation resistance.

## 1. Introduction

Metal additive manufacturing technology is a process for three-dimensional manufacturing of a product by stacking metal powders layer by layer using various energy sources and has the advantage of manufacturing the shape of a final product in a single process. Unlike other manufacturing processes, the metal additive manufacturing part cools very rapidly (approx. 10^7^ K/s); so, a specific microstructure is observed with suppressing crystal growth that affects mechanical and chemical characteristics [1,2,3,4,5,6]. Among various additive manufacturing technologies, the laser power bed fusion (LPBF) method has the advantage of manufacturing a relatively small but very complex part by depositing metal powders layer by layer in a powder bed and selectively melting metal powders using a laser as an energy source. This power bed fusion (PBF) technology suggests great potential in the metal processing field because it is possible to manufacture various products with complex shapes in a single process that were traditionally manufactured by cutting, machining, and brazing to manufacture various shapes of metals [7,8,9,10].

As metal 3D printing with a laser including PBF is repeated with fast melting and cooling of powder, it has a completely different microstructure, which yields dissimilar properties (e.g., tensile strength, elongation, wear, corrosion resistance) compared with materials manufactured through conventional working and heat treatment, even if the chemical composition is the same [11,12,13,14,15,16]. Since the characteristics of the known existing alloy material and the material manufactured by metal 3D printing are different from each other, new research needs to be conducted on the characteristics of the additively manufactured materials.

AISI 316L stainless steel (SS316L) is a representative austenitic stainless steel and has excellent corrosion resistance and machinability; so, it is widely used for engineering and medical applications such as flanges, pipelines, valves, surgical tools, and implants. In particular, due to its excellent corrosion resistance, it is extensively used as a manufacturing material having a complex shape by the LPBF process [17,18,19,20]. Among the various LPBF processes, SS316L manufactured by the selective laser melting (SLM) method shows fully dense and defect-free characteristics; so, printed SS316L parts could be applied without an additional post heat-treatment. For example, Li et al. [21] reported the results of a study to reduce bowling effect and microcrack according to process parameters by manufacturing near-fully-dense SS316L by SLM process. Liverani et al. [22] analyzed the microstructure and tensile properties of SS316 according to the SLM process variable. They concluded that optimizing the SLM process parameters showed superior ultimate tensile strength and elongation compared with SS316L alloys manufactured by traditional casting processes. Lee et al. [23,24] investigated the microstructure, tensile properties, and wear properties of SS316L produced by the SLM process according to the building direction. The tensile property and wear resistance are changed by the melt-pool boundary generated during the process and a sub-grain cell with a size of 1 μm or less, and it was reported that the alloy manufactured by the SLM process has similar mechanical properties compared with the cold-rolled material.

Thus, for SS316L alloy manufactured by the SLM process, plenty of research is being conducted on microstructure and mechanical properties according to process parameters, while chemical research on corrosion and oxidation properties is lacking. Ziętala et al. [25] studied the mechanical properties and corrosion resistance of SS316L produced by laser-engineered net shaping (LENS). They observed that about 5 μm sized elongated fine-grains of austenite and Cr- and Mo-enriched sub-grain boundary had a corrosion potential similar to conventionally manufactured SS316L, while having a relatively very low corrosion density. Lodhi et al. [26] evaluated corrosion properties in human serum, phosphate buffer saline, and 0.9 M NaCl environments to evaluate the biological properties of SLM-processed SS316L. They suggested that the reason the printed SS316L has a relatively high pitting corrosion resistance is because of the barrier behaviors of oxide films with a lack of MnS inclusion. Revilla et al. [27] compared and studied the corrosion properties of SS316L manufactured by two types of processes, which are SLM and laser metal deposition (LMD). In both processes, it was confirmed that an enriched amount of elements capable of improving corrosion resistance such as Cr, Ni, and Mo were present at the sub-grain boundary. It was revealed that the passivity was further improved because a finer microstructure was formed in alloys manufactured by the SLM process compared with the LMD technique. As such, the results of research on corrosion properties of metal vary depending on the additive manufacturing process, and since this research field is essential for practical application, various and many studies are required. In addition, materials produced through the PBF process have anisotropy according to the building direction, but the difference in corrosion resistance caused by anisotropy has not been studied in depth.

This study investigated the corrosion resistant characteristics of SLM-fabricated SS316L during the LPBF process in relation to building direction and post heat-treatment. In particular, stainless steels are vulnerable to pitting corrosion in Cl^-^-including environments (e.g., sea water); thus, the corrosion resistance of SLM-fabricated SS316L should be explored for practical applications. Changes in microstructure, corrosion resistance, and oxidation resistance of as-built specimens and samples with direct annealing heat-treatment without solution treatment were studied and compared with cold-rolled SS316L alloys with similar chemical compositions.

## 2. Materials and Methods

### 2.1. Preparation of Specimens

The SS316L samples examined in this study were fabricated by an LPBF-type metal 3D printer (Sodick Co., Ltd., OPM 250L, Kyoto, Japan). Gas-atomized spherical SS316L powder with an average particle diameter of 40 μm (OPM Laboratory Co., Ltd., OPM Stainless 316L) was used to fabricate the specimens. The detailed parameters of SLM used in this study are listed in Table 1. LPBF was performed under a nitrogen environment with an oxygen content of less than 1% to prevent oxidation. A so-called 90° rotate scanning strategy was used, i.e., the laser scanning lines are tilted by 90° between each layer. Further details of the material, including the microstructure, tensile property, and wear behavior relative to the building direction, were reported previously by a few of the present authors in [17,23,24,28].

**Figure 1:**
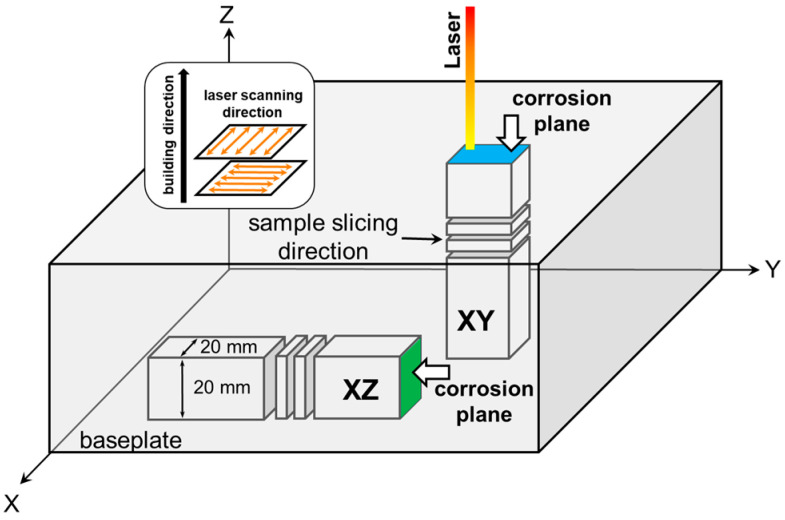
Schematic of sample geometries, scanning strategy, building direction, and corrosion test planes.

To evaluate the corrosion property of the SLMed SS316L samples, two types of specimens were built in different directions relative to the building orientation. The specimens were sliced from 20 × 20 × 100 mm^3^ blocks to achieve a thickness of 3 mm, each using wire electric discharge machining. The corrosion tests were carried out in the vertical and parallel directions to the baseplate, as shown in Figure 1. The specimens with a corrosion plane vertical to the building direction were designated as XY (blue area), whereas specimens with a corrosion plane horizontal to the building direction were designated as XZ (green area). Some specimens were directly annealed at 800 °C for 4 h, because SS316L is known to be fully annealed at 800 °C [29,30]. The post heat-treatment of the specimens was performed in a box-type laboratory furnace with an air atmosphere. The specimens were wrapped in a protective heat-treatment foil to prevent oxidation of the sample surface. The annealed XY and XZ samples were marked as AXY and AXZ, respectively. For comparison, a commercial-grade cold-rolled SS316L plate (thickness: 3 mm) was also used for the corrosion test. Specimens with dimensions of 20 × 20 mm^2^ were cut from the plate for the corrosion tests and these specimens were designated as CR. The chemical compositions of the OPM SS316L powder used for the LPBF process and the CR samples are given in Table 2.

### 2.2. Material Characterizations

The microstructures of the SS316L sample in this study were investigated using optical microscopy (OM, ECLIPSE LV150N, Nikon, Tokyo, Japan) and field-emission scanning electron microscopy (FE-SEM, JSM-7200F, Jeol Inc., Tokyo, Japan) with energy dispersive X-ray spectroscopy (EDS). The phase analysis was analyzed using X-ray diffraction (XRD, SmartLab, Rigaku, Tokyo, Japan) in the range of 2θ between 20° and 110° using the Cu Kα target (Kα = 1.5406 Å) under the condition of an acceleration voltage of 40 kV, current of 40 mA, and scanning speed of 1.5°/min.

The corrosion properties of each specimen were evaluated by a potentiodynamic polarization test in 3.5 wt.% NaCl solution (~pH 6.1) at room temperature. It was measured using a flat cell and potentiostat (VersaSTAT3, AMETEK, Berwyn, PA, USA). The Ag/AgCl (saturated KCl) and platinum mesh were used as reference and counter electrodes, respectively. The specimen was immersed in a 3.5 wt.% NaCl solution (~pH 6.8) for 20 min to stabilize the open circuit potential (OCP). The potential was scanned from −300 to 1500 mV vs. OCP at 2 mV/s rates.

The electrochemical impedance spectroscopy (EIS) measurements were obtained in potentiostatic mode using the same counter electrode and reference electrode in 3.5 wt.% NaCl solution. Measurements were investigated at the open circuit potential with the 10 mV AC amplitude over the frequency range from 10 kHz to 0.1 Hz. According to the characteristics of the Nyquist plot, an equivalent electrical circuit (EEC) was used to explain the corrosion properties of metals and their oxide films. By fitting data using EEC as the proposed model, Bode-impedance and Bode-phase angles were recorded to evaluate corrosion properties, and electrochemical parameters were obtained. 

X-ray photoelectron spectroscopy (XPS, NEXSA, Thermo Fisher Scientific, Waltham, MA, USA) was conducted to observe the oxidation state on the surface of specimen in relation to chemical compositions. All specimens were mechanically ground and polished down to SiC 2000 grit before the tests. Native oxide of each specimen was formed in an air atmosphere for 24 h. The binding energy was calibrated from the C-C contribution because of the C 1s adventitious carbon signal at 284.8 eV. The obtained data were handled using CasaXPS software [31].

To evaluate the high-temperature oxidation resistance, each sample was cut into cuboids with dimensions of 15 × 5 × 3 mm^3^. All specimens were mechanically ground and polished down to SiC 2000 grit before the tests. The high-temperature oxidation test was conducted in an air atmosphere at 700 °C for 24 h. In order to minimize error, the same 10 samples were used to measure the weight gain; then, averaged values were used. The thickness of the oxide layer was observed using FE-SEM.

## 3. Results and Discussion

**Figure 2:**
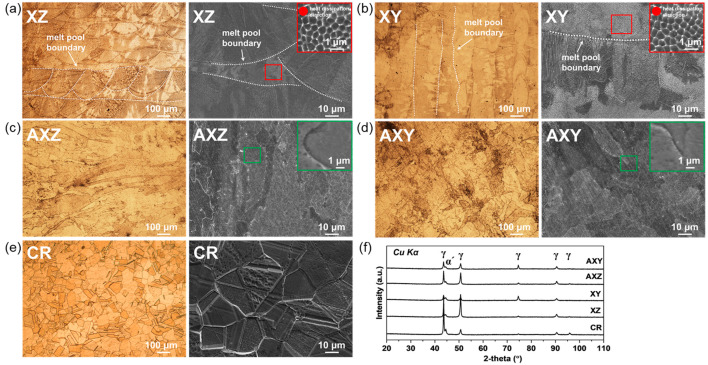
The microstructure images of each specimen: OM (left) and FE-SEM (right). Phase analysis of each specimen: (**a**) XZ, (**b**) XY, (**c**) AXZ, (**d**) AXY, (**e**) CR, (**f**) XRD pattern.

The microstructure and XRD pattern according to the building direction (XY and XZ) and heat-treatment (AXY and AXZ) of SS316L produced through the PBF process are compared with cold-rolled materials (CR) and shown in Figure 2. The melt-pool boundary can be observed in the XZ and XY as-built specimens (Figure 2a,b). As the XZ specimen has a plane parallel to the building direction, a semicircular melt-pool is observed, whereas the XY sample has a plane perpendicular to the building direction, so the melt-pool boundary is observed in a longitudinal direction along with a laser path during the printing process. Besides, the sub-grain cell with a size of 1 µm or less was clearly observed for both XZ and XY, which grows along the heat dissipation direction due to rapid solidification during the PBF process (red box in Figure 2a,b). It has been reported that such sub-grain cells improve mechanical properties because there are relatively many grain boundaries that inhibit the propagation of dislocation and deformation [32,33,34]. However, the melt-pool boundary was no longer observed in AXZ and AXY specimens after direct-aging heat-treatment, and sub-grain cells that improve mechanical strength also disappeared due to diffusion during the post heat-treatment (green box in Figure 2c,d) [35]. Even though such fine sub-grain cells disappear by post heat-treatment, the XRD pattern consists of austenite and alpha phase, and there were no phase changes by heat treatment (Figure 2f). Meanwhile, the CR sample has a smaller grain size than that of the SLMed SS316L (Figure 2e) [24], and the XRD pattern also consists of austenite and alpha phases. Therefore, it can be seen that the SLM-produced SS316L with sub-grain cell generated by rapid solidification has the same crystal structure as cold-rolled SS316L.

**Figure 3:**
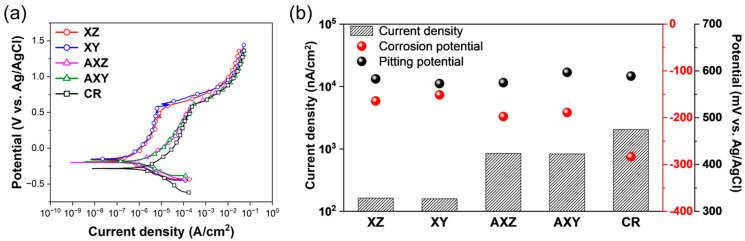
Corrosion resistance of each specimen in 3.5 wt.% NaCl. (**a**) Potentiodynamic polarization curves. (**b**) Comparison of corrosion and pitting potential, and current density.

The SS316L printed in one direction through the PBF process has a different arrangement of the melt pool and a different microstructure due to the laser scan direction, and thus, has mechanical anisotropy according to the building direction of the material [23,24,36,37]. The SS316L alloy is very important in applications for corrosion resistance as well as mechanical properties; so, it is necessary to evaluate it compared with the cold-rolled SS316L. Therefore, a potentiodynamic polarization test was performed in a 3.5 wt.% NaCl solution to evaluate the corrosion resistance of each specimen. The results are presented in Figure 3. The corrosion current densities of XZ and XY specimens were 163 and 159 nA/cm^2^, respectively, and the corrosion potentials were −160 and −150 mV, respectively, showing similar results. Furthermore, the current density and pitting potential in the passive region were also similar. These results indicate that the anisotropy in corrosion resistance by the laser scan direction during the PBF process is insignificant, while the mechanical properties show anisotropy according to the morphology and arrangement of the melt pool, which are determined by the laser scan direction [23,24,37,38,39]. 

For cold-rolled SS316L, the corrosion current density is higher than that of XZ and XY, and the corrosion potential is lower than that of XZ and XY samples; further, the current density of CR SS316L in the passive region was higher than that of SS316L produced by the PBF process. This result indicates that the corrosion resistance of SS316L fabricated by the PBF method is superior to cold-rolled SS316L due to sub-grain cells formed during the PBF process. Partial segregation of Cr, Ni, and Mo elements that can improve corrosion resistance is generated at the boundary of sub-grain cells caused by fast cooling, and the sub-grain boundary acts as a barrier to suppress corrosion and has superior corrosion resistance [25,27,38,40]. The AXZ and AXY specimens produced by annealing heat treatment decreased their corrosion potential to −200 mV for AXZ and −190 mV for AXY compared with the as-built sample and increased their corrosion current density to 848 nA/cm^2^ for AXZ and 831 nA/cm^2^ for AXY. The passivation current density has also increased considerably. These results show that the corrosion resistance of SS316L is reduced by annealing, accelerating a diffusion of alloying elements. In particular, the elimination of sub-grain boundary enriched with Ni, Cr, and Mo inhibiting the corrosion propagation is critical for corrosion resistance of SS316L fabricated by PBF. Nevertheless, since the current density of AXZ and AXY is lower than the CR specimen and the corrosion potential of AXZ and AXY is higher than the CR in the corrosion current density and passivation region, it is known that the corrosion resistance is better than that of the existing cold-rolled SS316L. Moreover, from the similar corrosion current density and corrosion potential of AXZ and AXY in the current density and passivation region, it can be seen that anisotropy of corrosion resistance is not formed by heat treatment. On the other hand, in the case of the pitting potential, which means the start of the pitting, all samples showed a pitting potential of about 590 mV, which was similar. This is attributed to the fact that the SS316L materials analyzed in this study did not cause precipitation and structural heterogeneity in the microstructure, which could be the initiation point of pitting corrosion.

**Figure 4:**
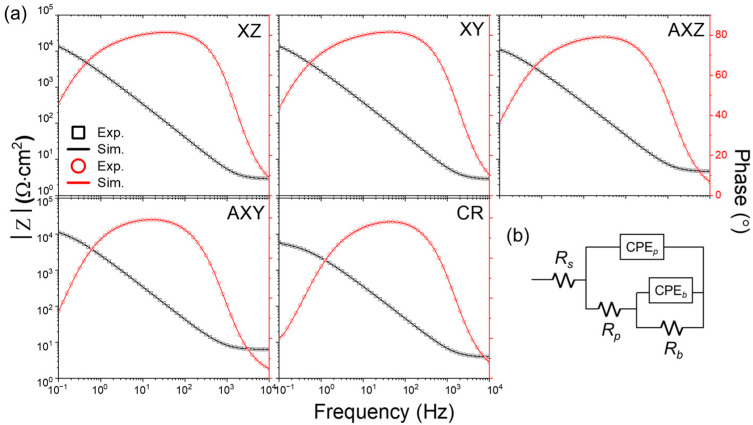
(**a**) Bode spectra for each specimen in 3.5 wt.% NaCl solution. (**b**) Equivalent circuit for data fitting.

The passive oxide film contributes to the excellent corrosion resistance of stainless steel. The characteristics of the passive film of the PBF-processed SS316L alloy affecting corrosion resistance were evaluated through electrochemical impedance spectroscopy (EIS) analysis, and the results are shown in Figure 4a as a Bode plot. The equivalent circuit for EIS measurement is shown in Figure 4b, and the fitting results are summarized in Table 3. The physical meaning of *R_s_*, *R_p_*, and *R_b_* are resistance of the electrolyte, redox transformation of corrosion product on oxide surface, and charge transfer reaction at metal/oxide interface, respectively. CPE*_p_* and CPE*_b_* represent the capacitance of the double layer on oxide surface and barrier oxide layer. In addition, *n_p_* and *n_b_* correspond to exponent of CPE*_p_* and CPE*_b_*, respectively. A higher value of *R_p_* + *R_b_* implies a higher corrosion resistance to the corrosive media. However, the *R_b_* is significantly higher than *R_p_* by more than 2 orders of magnitude, and the total corrosion resistance is mostly determined by the *R_b_*. 

The *R_b_* of the as-built materials XZ and XY were 28.61 and 27.73 kΩ cm^2^, respectively, 50% or higher than that of the *R_b_* of the heat-treated AXZ (17.22 kΩ cm^2^) and AXY (15.94 kΩ cm^2^), respectively. Furthermore, the *R_b_* of as-built XZ and XY SS316L shows four times higher than *R_b_* (6.67 kΩ cm^2^) of cold-rolled SS316L and two times or higher than that of CR materials in heat-treated SLM-processed SS316L. In the EIS analysis, there was no significant difference in resistance according to the building direction. The higher the *R_b_*, the lower the reaction at the oxide/metal interface; so, the redox reaction of the corrosion product on the oxide surface decreases, resulting in a higher *R_p_* value. This high *R_b_* is related to the surface passivation film of SS316L alloy and, in a chemically stable state, the electrochemical reaction that causes corrosion at the oxide/metal interface is suppressed; hence, the corrosion rate is low. Therefore, it can be seen that the results of EIS measurement on as-built SS316L and model fitting are well matched with the results of the potentiodynamic polarization test.

**Figure 5:**
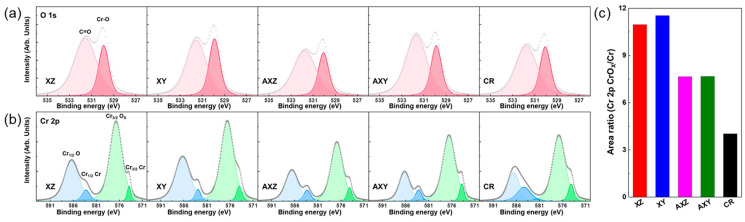
XPS core level spectra of (**a**) O 1s and (**b**) Cr 2p of XZ, XY, AXZ, AXY, and CR samples. The colored line shows peak-deconvoluted bands. The Me-O (530.6 eV) related peak of XZ and XY showed a higher oxide ratio compared with the other samples. (**c**) Represents fitted peak area ratio of oxide-related and metal-related peaks of Cr 2p. XZ and XY have a higher oxide formation ratio compared with AXZ, AXY, and CR.

The corrosion resistance of SS316L fabricated by PBF is affected by the resistance of passive oxide layer on metal surface. The difference in the oxide layer of 316L used in this study can be analyzed by XPS. Figure 5 represents XPS core level spectra of (a) O 1s and (b) Cr 2p of XZ, XY, AXZ, AXY, and CR. The two peaks can be found in O 1s spectra at 530.6 eV and 531.6 eV, which are related to metal oxides and carboxyl groups by adsorbed gas layer on oxide [41,42]. The XPS core level spectra of Cr 2p can be deconvoluted into two peaks for chromium metal (583.5 eV (Cr1/2) and 573.9 (Cr3/2)) and chromium oxide (Cr_2_O_3_, 586.8 eV (Cr1/2) and 576.5 eV (Cr3/2)) bonding. The more Cr_2_O_3_ on stainless steel indicates a better passivation layer against corrosive environments. Therefore, the area ratio of Cr_2_O_3_/Cr in Cr 2p was estimated and is shown in Figure 5c. The area ratios of as-built SS316L (XZ and ZY) are ~11, which is higher than the cases with annealing (AXZ and AXY, ~7.7) and CR (~4.0). Similar area ratios between XZ and XY, and between AXZ and AXY, indicate that anisotropy in the oxide formation of SS316L fabricated by PBF was not found. Moreover, the higher area ratio of as-fabricated SS316L indicates that the microstructure with a sub-grain cell is effective to create a highly concentrated Cr_2_O_3_ layer on stainless steel, which is chemically stable in corrosive environments, so that more passivity against corrosion is obtained. For these reasons, as-built 316L samples (XZ and XY) with the highest ratio of Cr_2_O_3_/Cr have the most robust corrosion resistance. Due to the dissolution of the sub-grain cell of as-built 316L (XZ and XY) by the thermal annealing, the ratio of Cr_2_O_3_/Cr is decreased, indicating a diminished passivity of the oxide layer. Therefore, the corrosion resistance of AXZ and AXY is lower than those of as-built 316L samples. Moreover, the CR is created with various processes to uniformly distribute alloying elements and remove local chemical compositional irregularity; thus, it shows the lowest Cr_2_O_3_/Cr ratio compared with 316L fabricated by PBF, which causes local irregularity in the microstructure. Therefore, the CR that commercialized SS316L has a relatively low corrosion resistance compared with SS316L fabricated by PBF.

**Figure 6:**
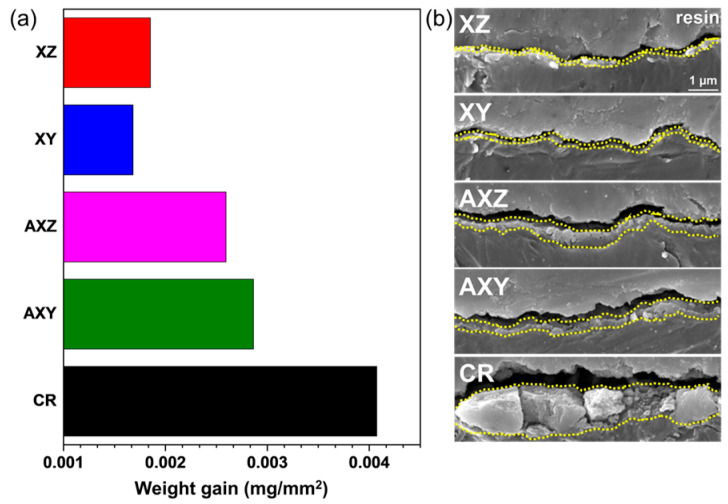
High-temperature oxidation of each specimen at 700 °C for 24 h. (**a**) Weight gain and (**b**) cross-sectional morphology of oxide layer.

SS316L is known to have high corrosion resistance as well as high resistance to surface oxidation. To confirm its applicability at a high temperature in an air atmosphere, the high-temperature oxidation behaviors of the SLM-fabricated alloy and the cold-rolled materials were compared. Each specimen was exposed to 700 °C for 24 h in an air atmosphere. The results of the increase in weight and cross-sectional microstructure observation due to high-temperature oxidation are shown in Figure 6a,b, respectively. The weight gains (~0.0017 mg/mm^2^) of XY and XZ specimens after the oxidation test are similar and very little oxidation occurred; so, the cross-sectional oxide layers were not clearly distinguished in the SEM observation. This means that there is no change in the high-temperature oxidation characteristics due to the arrangement of microstructures along the building direction. When the sub-grain cell of SLMed SS316L was removed through the heat treatment, the weight increase due to oxidation on the surface was ~0.0027 mg/mm^2^, an increase of ~50% compared with the specimen before heat treatment, and a thin oxide film was observed in the cross-sectional area.

In the case of CR alloys, the weight increase due to oxidation is ~0.0041 mg/mm^2^, which is 2.4 times higher than that of as-built SS316L (XZ and XY) and about 1.5 times higher than that of direct-annealed SS316L (AXZ and AXY), respectively. In addition, a very thick oxide layer can be observed with SEM analysis. High oxidation resistance of as-built SS316L is attributed to the locally concentrated antioxidant elements such as Cr, Ni, and Mo in sub-grain cells, which inhibit additional surface oxidation, and it is very beneficial to form a large portion of chemically stable Cr_2_O_3_ on the surface. However, the sub-grain cell is removed by post heat-treatment, and high oxidation resistance is also degraded.

The sub-grain cell is generated by locally concentrating alloy elements as molten metal is rapidly cooled, and is the most representative characteristic of the alloy materials printed with PBF and direct energy deposition (DED) processes [43,44,45,46]. The sub-grain cell enables higher mechanical properties and workability than general cold-rolled materials [17,28,47,48]. Additionally, this study confirmed that this sub-grain cell contributes to the formation of a high amount of Cr_2_O_3_ that can increase the chemical and electrochemical stability of SS316L. As a result, SS316L manufactured by the PBF process showed higher corrosion and oxidation resistance than cold-rolled materials. However, due to the heat treatment, the sub-grain cell was removed, thereby reducing corrosion and oxidation resistance, and it has been reported that mechanical properties are also reduced by the elimination of sub-grain cells [23,24]. Therefore, there will be a limit to the application temperature capable of maintaining the mechanical/chemical properties of SLMed SS316L, and further research is needed to investigate this. The thermal stability of sub-grain cells of stainless steel formed through additive manufacturing and research to improve them should also be conducted in the near future.

## 4. Conclusions

A SS316L alloy made by the PBF process has a microstructure called a sub-grain cell, which is generated by the local concentration of alloy elements. This microstructure is very effective in forming chemically stable Cr_2_O_3_ at a high concentration on the surface of SS316L. As a result, the SLM-fabricated SS316L materials with sub-grain cells have excellent corrosion and oxidation resistance due to their higher chemical stability compared with conventional cold-rolled SS316L. However, due to the post heat-treatment, an amount of the local alloy element concentrates at the sub-grain cell are removed, and the stable amount of Cr_2_O_3_ for the oxide film formed on the surface is reduced, thereby reducing corrosion and oxidation resistance. In addition, unlike mechanical properties, corrosion and oxidation resistance are independent to the building direction of PBF. Accordingly, the SLMed SS316L material with excellent corrosion resistance and oxidation resistance must be applied within a temperature range in which sub-grain cells are not diminished to maintain its performance.

## Figures and Tables

**Table 1:** Parameters used in the selective laser melting process.

Parameter	Setting
Laser power	370 W
Scanning speed	800 mm/s
Hatch spacing	0.1 mm
Lamination thickness	0.05 mm

**Table 2:** Chemical compositions of OPM SS316L powder and cold-rolled specimens.

Elements (wt.%)	Fe	Cr	Ni	Mo	Mn	Si	C	P	S	N
OPM powder	Bal.	16.91	10.25	2.11	1.24	0.75	0.05	0.03	0.02	0.02
cold-rolled	Bal.	16.93	10.11	2.09	1.36	0.47	0.04	0.03	0.01	0.03

**Table 3:** Fitted data for parameter of equivalent circuit.

Sample Name	*R_s_*, Ω cm^2^	CPE*_p_*, µF/cm^2^	*n*_p_	*R_p_*, Ω cm^2^	CPE*_b_*, µF/cm^2^	*n*_b_	*R_b_*, kΩ cm^2^
XZ	2.91	27.76	0.99	90.34	44.11	0.69	28.61
XY	2.84	28.82	0.98	88.89	46.64	0.68	27.73
AXZ	3.65	38.43	0.99	40.47	53.76	0.70	17.22
AXY	3.31	35.35	0.98	56.46	55.18	0.71	15.94
CR	3.93	48.51	0.99	31.79	64.30	0.69	6.67
